# Keap1 Cystenine 151 as a Potential Target for Artemisitene-Induced Nrf2 Activation

**DOI:** 10.1155/2019/5198138

**Published:** 2019-10-15

**Authors:** Shanshan Liu, Shengmei Xu, Renrong Wei, Zhizhong Cui, Xiaoyun Wu, Renxiong Wei, Li Xie, Yingye Zhou, Wenjuan Li, Weimin Chen

**Affiliations:** ^1^Second Clinical Medical College, Guangzhou University of Chinese Medicine, 232 Waihuan Road East, Guangzhou, Guangdong 510006, China; ^2^Center for Regenerative and Translational Medicine, Guangdong Provincial Academy of Chinese Medical Sciences, The Second Affiliated Hospital of Guangzhou University of Chinese Medicine, Guangzhou, Guangdong 510006, China; ^3^Sino-French Hoffmann Institute, School of Basic Sciences, Guangzhou Medical University, Guangzhou, Guangdong 511436, China; ^4^Department of Pharmacy, The Second Affiliated Hospital, Guangzhou University of Chinese Medicine, Guangzhou, Guangdong 510006, China; ^5^Shunde Hospital, Southern Medical University, The First People's Hospital of Shunde Foshan, Foshan, Guangdong 528000, China; ^6^Hainan Medical University, Haikou, Hainan 571199, China

## Abstract

Artemisitene (ATT) activates the nuclear factor (erythroid-derived 2)-like 2 (Nrf2) by increasing its stabilization and reducing ubiquitination. The cysteine (Cys) residues of the cytosolic Nrf2 repressor Kelch-like ECH-associated protein-1 (Keap1) function as redox sensors and may be crucial in activating Nrf2. To determine whether ATT-induced Nrf2 activation is dependent on the modification of Keap1 and to elucidate the underlying mechanism, we transfected cell lines with six different Keap1 mutant constructs, each with a Cys (−77, −151, −257, −273, −288, and −297) to Ser substitution. Only the Cys151Ser mutant prevented ATT-mediated activation of Nrf2, indicating that the Cys151 residue of Keap1 likely interacts with ATT and is essential for Nrf2 stabilization and transcription of downstream genes. Our finding provides a pharmacological basis for using artemisitene against oxidative stress-related diseases.

## 1. Introduction

Nuclear factor (erythroid-derived 2)-like 2 (Nrf2) is a transcription factor that activates multiple genes, including drug transporters, antioxidant enzymes, and phase II detoxifying enzymes like heme oxygenase (HO-1)[1, 2]. The Nrf2 signaling pathway is tightly regulated [3–7] by Keap1, the substrate adaptor protein of the cul3-dependent E3 ubiquitin ligase, which specifically binds to Nrf2 and results in the latter's polyubiquitination and cytoplasmic retention [8–11]. Keap1-dependent ubiquitination of Nrf2 is inhibited by oxidative stress, as well as the chemical inducers of Nrf2, which activates the Nrf2-dependent downstream protective genes [9]. Studies show that modification of specific cysteine (Cys) residues in Keap1 plays a critical role in the oxidative stress or chemical-induced activation of Nrf2 [4, 12]. Most chemical inducers of Nrf2 covalently modify the Cys151 residue of Keap1, which is also the target of reactive oxygen species (ROS) and other electrophiles. Both covalent and oxidative changes in Cys151 destabilize the Keap1-Cul3 interaction and subsequently activate the Nrf2-dependent downstream genes [13]. Site-directed mutagenesis on the conserved Cys residues of Keap1 by later studies showed that Cys77, Cys151, Cys257, Cys273, Cys288, and Cys293 residues are also critical for Nrf2 activation [9, 12–15].

Several phytochemicals have recently been shown to activate Nrf2. Artemisitene (ATT), a semisynthetic derivative of the sesquiterpene artemisinin isolated from *Artermisia annua* [1, 5], can activate Nrf2 by blocking its ubiquitination and increasing its stability [16]. However, the underlying molecular mechanism is still unclear. In the present study, we found that ATT activated the Nrf2-dependent pathway by covalently modifying the Cys151 of Keap1, which provides a strong pharmacological basis for its future applications in oxidative stress-related diseases.

## 2. Methods

### 2.1. Cell Culture and Reagents

Cos-1, A549, and 293T cell lines were obtained from the American Type Culture Collection (ATCC; Manassas, VA, USA). All lines were checked for mycoplasma contamination at least once a month using the mycoplasma PCR detection kit. The cells were cultured in 10% fetal bovine serum- (FBS-) supplemented DMEM at 37°C under 5% CO_2_. Tert-butylhydroquinone (tBHQ) was purchased from Sigma Chemical (St. Louis, MO, USA) and ATT from Tianjin Silan Technology Co. Ltd. The cells from the 2^nd^ to 5^th^ passages were used for the assays.

### 2.2. Western Blotting

The cells were washed with cold PBS and lysed on ice with NP-40 cell-lysis buffer supplemented with 2% 2-mercaptoethanol, 50 mM DTT, and 1% Protease Inhibitor Cocktail. The lysates were cleared by centrifuging for 15 minutes at 13,000 rpm, and the protein content of the supernatants was evaluated using the BCA assay. Equal amount of proteins per sample were denatured in the sample loading buffer by boiling for 5 min, resolved in 7.5% and 10% SDS-polyacrylamide gels, and then transferred onto polyvinylidene difluoride (PVDF) membranes. The latter was blocked with 5% skimmed milk in TBST (TRIS-buffered saline with 0.1% Tween-20) at room temperature for one hour and incubated overnight at 4°C with the primary antibodies against Nrf2 (1 : 1000; Abcam, Cambridge, UK, ab76026, Rabbit monoclonal), Keap1 (1 : 500; Proteintech, IL, USA, 10503-2-AP, Rabbit monoclonal), GAPDH (1 : 500; Cell Signaling, OH, USA), and HO-1 (1 : 1000; Abcam, Cambridge, UK, ab13243, Rabbit monoclonal). The blots were rinsed thrice with TBST and incubated with the horseradish peroxidase-conjugated secondary antibodies at room temperature for 1 hour. After washing thrice with TBST, the bands were developed using an ECL substrate solution (Super Signal™ West Dura Extended Duration Substrate, Thermo fisher) for 1 min [17]. The grey value of proteins was measured by ImageJ (NIH, Bethesda, MD, USA). Averages of three independent experiments were presented as the final data [18].

### 2.3. Plasmids and siRNA

The plasmids pcDNA 3.0, pcDNA 3.0-keap1-wt, pcDNA3.0-Nrf2, and pGL4.22-ARE-lucferase were gifts from Donna D. Zhang (University of Arizona). Site-directed mutagenesis of Cys77, Cys151, Cys257, Cys273, Cys288, and Cys 293 in pcDNA3.0-keap1 was conducted using the Quick-Change Site-Directed Mutagenesis Kit (Agilent Technologies, Santa Clara, CA, USA) and Mut Express II Fast Mutagenesis KitV2 (Vazyme, Nanjing, China). The resulting plasmids—pcDNA3.0-keap1-C77s, pcDNA3.0-keap1-C151s, pcDNA3.0-keap1-C257s, pcDNA3.0-keap1-C273s, pcDNA3.0-keap1-C288s, and pcDNA3.0-keap1-C293s—were verified by gene sequencing. The siRNA targeting Keap1 was purchased from Gena Pharma (China).

### 2.4. Transfection

The cells were seeded in 6-well plates and transfected with the requisite plasmids diluted 1 : 1 in Lipofectamine® 3000 (Thermo Fisher Scientific Life Sciences, Waltham, MA, USA) according to the manufacturer's instructions [19].

### 2.5. Luciferase Reporter Gene Assay

Cos-1 cells were cotransfected with 40 ng each of the ARE-luciferase and Renilla luciferase expression plasmids, 80 ng of the wild or mutant-type Keap1 plasmid, and 80 ng Nrf2 plasmid using lipofectamine 3000. Forty-eight hours after transfection, the cells were treated with 7 *μ*M ATT for 24 h. ARE-luciferase activity was quantified using the dual luciferase reporter assay system (Promega, CA, USA) and normalized to that of Renilla luciferase.

### 2.6. Detection of Intracellular ROS Levels

Intracellular ROS levels were detected using the oxidation-sensitive fluorescent probe DCFH-DA. After treatment with varying doses (1 *μ*M, 4 *μ*M, and 7 *μ*M) ATT or 0.05% DMSO with or without 10 mM ROSUP for 6 h or 16 h, the cells were washed twice with phosphate-buffered saline (PBS). After incubating with 10 *μ*M DCFH-DA at 37°C for 20 min, the fluorescence intensity of the oxidized 2,7-dichlorofluorescein (DCF) was detected on a flow cytometer (Becton Dickinson). For each sample, 10000 events were analyzed.

## 3. Results

### 3.1. ATT Activates the Nrf2 Signaling Pathway in a Keap1-Dependent Manner

**Figure 1:**
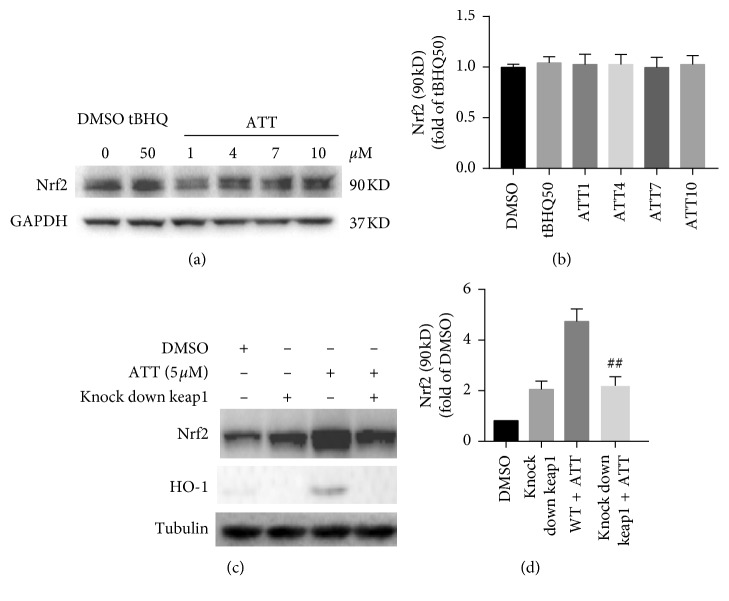
ATT-dependent Nrf2 upregulation requires functional Keap1. A549 cells (a) and WT/Keap1-knockdown MB231 cells (c) were exposed to predetermined ATT doses for 16 hours. Immunoblots show the levels of Nrf2, HO1, tubulin, and GAPDH. Control cells were treated with 50 *μ*M tBHQ. The quantization of Nrf2 in the A549 and MB231 cells are shown in (b) and (d), respectively, (all values are represented as mean ± SD, *n* = 3, ^##^*P* < 0.01).

To determine whether ATT activates Nrf2 in a Keap1-dependent manner, we treated MB231 cells and A549 cells that harbor a mutated form of Keap1, with ATT. While no changes were seen in the Nrf2 levels in A549 cells after ATT treatment (Figures 1(a) and 1(b)), ATT upregulated Nrf2 in the MB231 cells (Figures 1(c) and 1(d)). However, knocking down Keap1 in the MB231 cells abrogated the effect of ATT (Figures 1(c) and 1(d)), which clearly indicated that ATT-induced Nrf2 activation is dependent on Keap1.

### 3.2. ATT Targets Keap1 at Cys151

**Figure 2:**
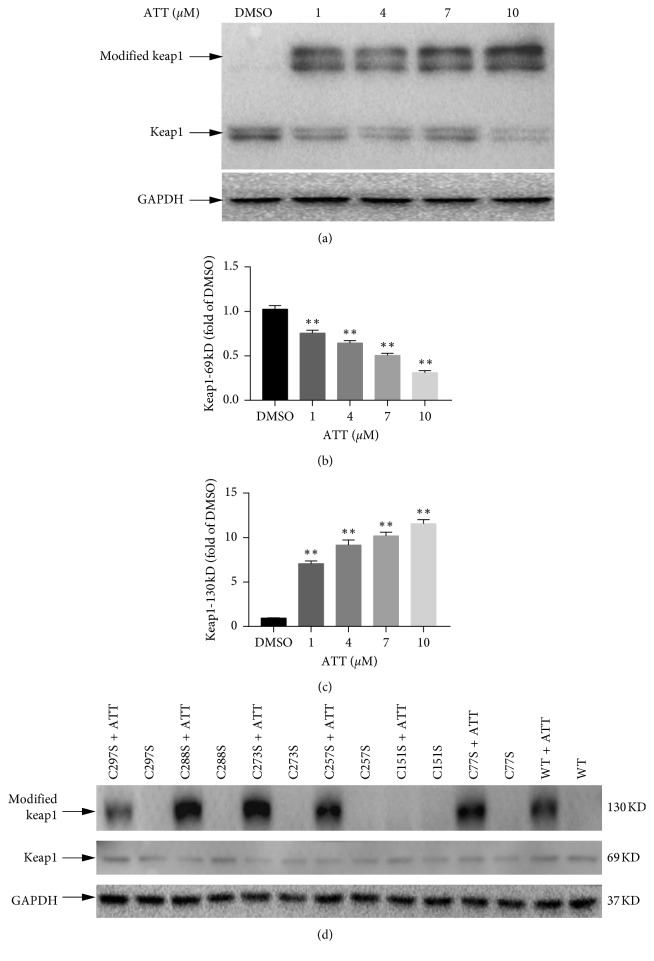
Cysteine 151 is required for ATT-induced modification to Keap1. (a) COS1 cells were transfected with Keap1-expressing plasmid and treated with varying doses of ATT for 16 hours. Immunoblots show Keap1 and GAPDH levels. (d) COS-1 cells were transfected with vectors expressing either mutant Keap1 (lanes 1 to 12) or wild-type (WT) Keap1 (lanes 13–14) and treated with either 7 *μ*M ATT (+) or DMSO (−) for 16 hours. (b-c) Comparison of the expression levels of modified Keap1 and keap1-69KD at different does of ATT. All values represent mean ± SD of three experiments; ^*∗*^*P* < 0.05; ^*∗∗*^*P* < 0.01.

The cysteine residues in Keap1 act as redox sensors, and most known Nrf2 inducers such as tBHQ can induce covalent modification of Cys151 or other cysteine residues of Keap1 [3, 13]. To determine whether ATT has a similar mechanistic basis, we constructed several Keap1 mutants, each with a single Cys to Ser mutation at residues 77, 151, 257, 273, 288, or 297. Cos-1 cells were transfected with wild-type or mutated keap1 constructs and treated with varying doses of ATT. A 68 KD band corresponding to the WT Keap1 protein was observed in the untreated control lysates, and a larger 120 KD band was also observed in the ATT-treated lysates (Figure 2(a)), the intensity of which was dependent on the ATT dose (Figures 2(a)–2(c)). ATT treatment induced the larger band in all Keap1 mutant (77Cys-Ser, 257Cys-Ser, 273Cys-Ser, 288cys-Ser, and 297Cys-Ser) lysates, except the 151Cys-Ser mutant. Taken together, Keap1 is covalently modified by ATT at the Cys151 site (Figure 2(d)).

### 3.3. Keap1-Cys151 Is Essential for Nrf2 Activation by ATT

**Figure 3:**
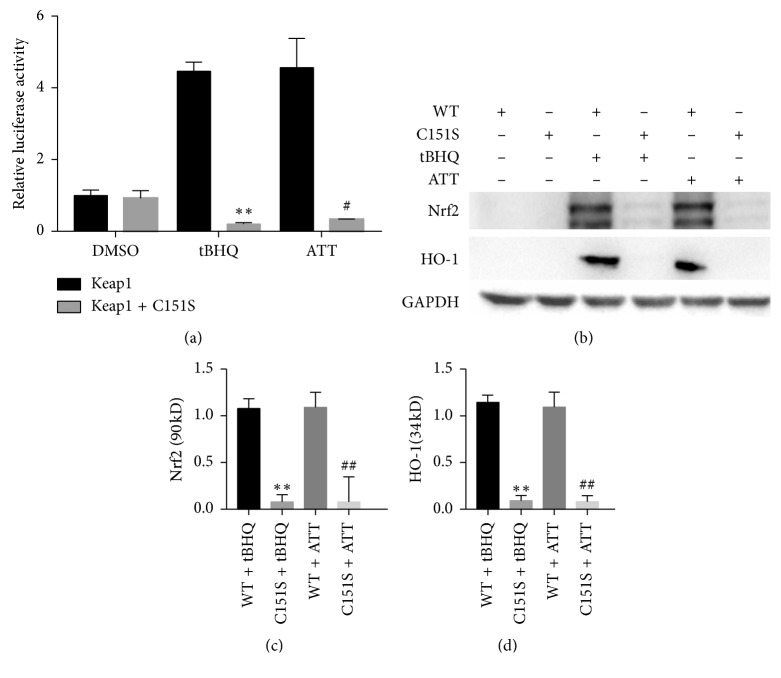
ATT activates Nrf2 signaling pathway by modifying Keap1 at C151. (a) COS-1 cells were cotransfected with ARE-luciferase, Renilla luciferase, WT or mutant Keap1, and Nrf2 expression plasmids and treated with 7 *μ*M ATT after 48 hours. Relative firefly luciferase activity in cell expressing WT and mutant Keap1 was calculated and normalized against that of Renilla. Data are expressed as mean ± SD of three experiments. ^*∗*^*P* < 0.05 and ^*∗∗*^*P* < 0.01 compared to WT Keap1 and DMSO, respectively; ^#^*P* < 0.05 and ^##^*P* < 0.01 compared to Keap1-C151S and DMSO, respectively. (b) Immunoblots showing Nrf2 and HO-1 levels in the above cells and the quantification of Nrf2 (c) and HO-1 (d). All values are represented as mean ± SD of three experiments. ^*∗*^*P* < 0.05 and ^*∗∗*^*P* < 0.01 compared to untreated and tBHQ, respectively; ^#^*P* < 0.05 and ^##^*P* < 0.01 compared to WT and ATT, respectively.

**Figure 4:**
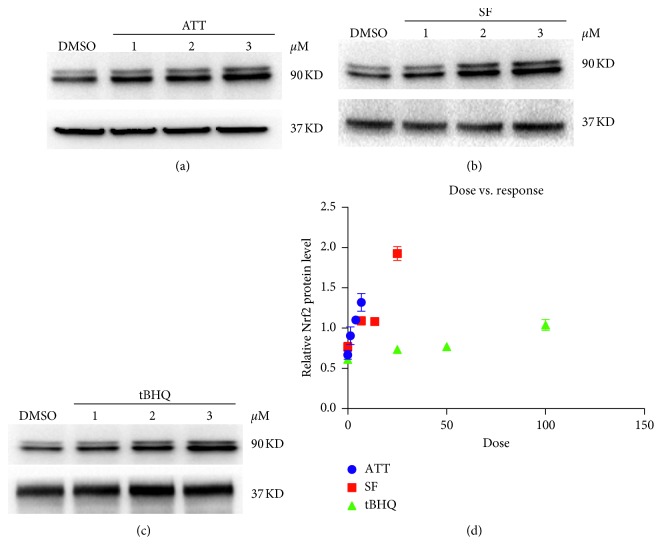
ATT is effective at low doses. Immunoblots showing Nrf2 levels in cells treated with 0.05% DMSO (lanes 1): 1 *μ*M, 3 *μ*M, and 5 *μ*M ATT (a); 5 *μ*M, 10 *μ*M, and 25 *μ*M sulforaphane (b); or 25 *μ*M, 50 *μ*M, and 100 *μ*M tBHQ (c). (d) Scatter plot showing the dose responsiveness in the differentially treated cells.

To determine whether Keap1-Cys151 is essential for Nrf2 activation, the Cos-1 cells were cotransfected with WT/C151S Keap1, Nrf2, and Renilla and ARE-luciferase plasmids. As expected, ATT significantly increased the ARE-dependent transcriptional activity in WT-Keap1 transfected cells compared to the untreated control. However, ATT had no effect on ARE-dependent transcriptional activity in the cells expressing Keap1-C151S (Figure 3(a)), which also showed decreased Nrf2-dependent transcriptional activity compared to cells expressing the WT Keap1 (Figure 3(a)). This indicated a loss of activation phenotype conferred by the C151S mutation and that Keap1-Cys151 is essential for Nrf2 activation by ATT. Furthermore, we explored whether Keap1-Cys151 is also essential for ATT-mediated Nrf2 stabilization. The Keap1-C151S cells expressed significantly lower Nrf2 levels after ATT treatment compared to the WT cells. Accordingly, ATT did not activate the Nrf2-dependent downstream gene HO-1 in the cells harboring the Keap1-C151S mutant (Figures 3(b)–3(d)). Taken together, Keap1-Cys151 is necessary for ATT-dependent Nrf2 stabilization and downstream transcriptional activation (Figure 4).

### 3.4. ATT Activates the Nrf2 Pathway at Low Dose

Nrf2 levels were significantly increased following different chemical inducers compared to that in the untreated sample. However, while tBHQ and sulforaphane were only effective at high doses, ATT increased Nrf2 levels at low doses ranging from 1 to 5 *μ*M. In addition, 3 *μ*M ATT was nearly as effective as 10 *μ*M SF and 100 *μ*M tBHQ, indicating greater sensitivity of ATT.

### 3.5. ATT Has No Effect on the Oxidative Stress

**Figure 5:**
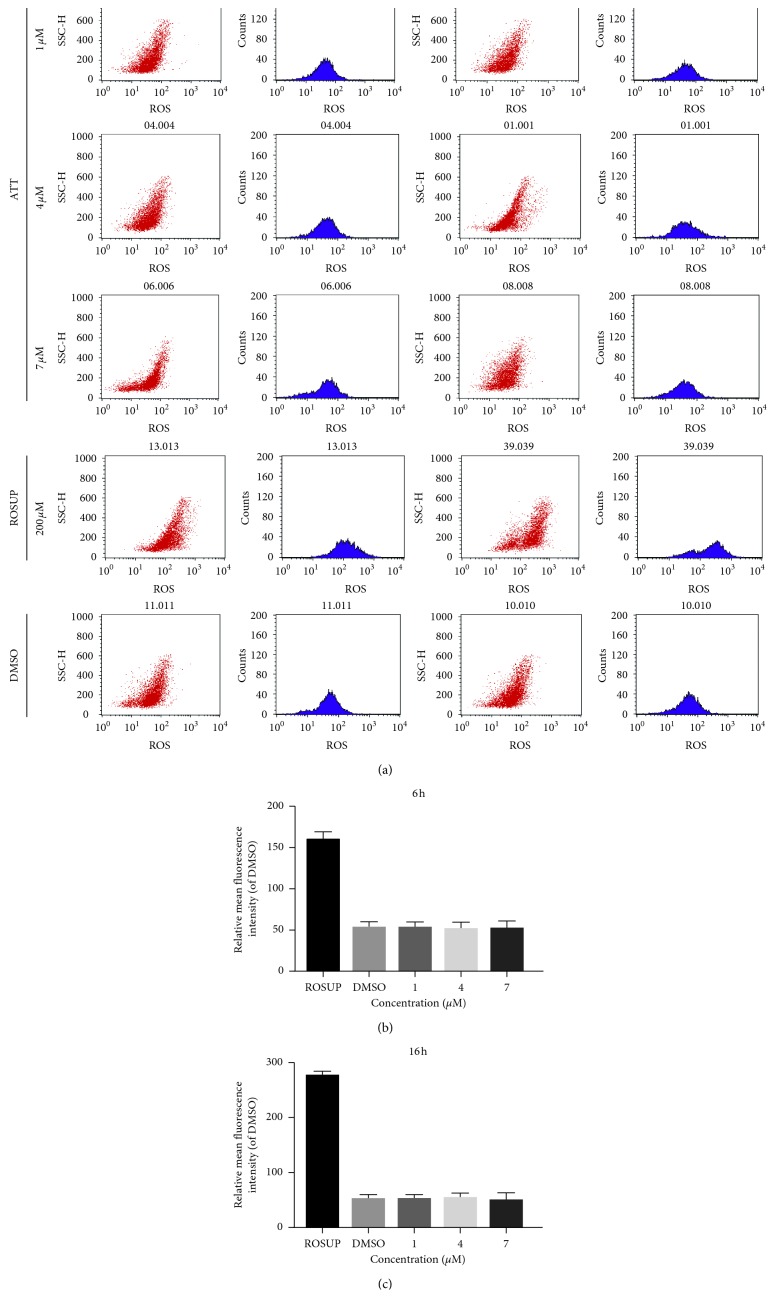
ATT does not affect intracellular ROS levels. Cells were treated with 50 *μ*M ROSUP, 0.05% DMSO, or 1 *μ*M, 4 *μ*M, and 7 *μ*M ATT for 6 h or 16 h. Histograms show fluorescence intensities of oxidized DCF in the differentially treated cells and are compared in the bar graphs.

As shown in Figure 5, ATT treatment did not have any significant effect on the fluorescence intensity of the oxidized DCF compared to the untreated controls. Thus, ATT does not increase ROS production and cannot active Nrf2 by inducing oxidative stress.

## 4. Discussion

Nrf2 activation by specific chemicals prevents or inhibits the progression of oxidative stress-related disorders, including diabetes, cancer, cardiovascular diseases, neurodegenerative diseases, chronic obstructive pulmonary disease, pulmonary fibrosis, and inflammation in experimental animal models. ATT, a derivative of the antimalarial agent artemisinin, was recently identified as a novel Nrf2 activator [16]. In this study, we further elucidated the mechanisms underlying ATT-mediated activation of Nrf2 and its downstream pathway, which provide a crucial pharmacological basis for its future clinical applications.

Nrf2 is repressed by the Keap1 protein under physiological conditions, which is reversed upon exposure to reactive chemicals, oxidative stress, or chemopreventive agents. Zhang and Hannink first reported that Keap1 targets Nrf2 for ubiquitination and proteasome-dependent degradation through its Cys residues, and C151 in particular is required for stabilizing Nrf2 in response to tBHQ- and sulforaphane-induced oxidative stress [12]. The Cys residues in Keap1, especially C151 and C288, act as redox sensors for activation of Nrf2 [13] by undergoing covalent modification. In this study, we confirmed that covalent modification of the Cys151 residue is essential for Nrf2 activation by ATT. Based on the structural comparison between artemisitene and artemisinin, we concluded that the distinct ene bond in the former is critical for Cys151-dependent Nrf2 activation by ATT [16]. Taken together, an ATT-based therapeutic approach targeting Nrf2 is a promising strategy to control diseases associated with oxidative stress.

## Data Availability

The data used to support the findings of this study are available from the corresponding author upon request.
